# Burnout subtypes in the German working population: Differentiation by symptomatology, work-related factors and structural impairment according to the OPD

**DOI:** 10.1371/journal.pone.0352860

**Published:** 2026-07-06

**Authors:** Julia Perlinger, Johannes C. Ehrenthal, Christiane Montag, Thomas Kretschmar

**Affiliations:** 1 Department of Psychiatry and Neurosciences, Charité - Universitätsmedizin Berlin, Corporate Member of Freie Universität Berlin and Humboldt-Universität zu Berlin, Berlin, Germany; 2 Department of Psychology, Universität zu Köln, Cologne, North Rhine-Westphalia, Germany; 3 Mind-Institute, Berlin, Germany; Medical University of Vienna, AUSTRIA

## Abstract

Farber’s typological approach expands burnout models by identifying three burnout subtypes: frenetic, underchallenged, and worn-out. Despite its potential, research on this approach is limited, especially in the German working population. This study translated and validated the Burnout Questionnaire for Clinical Subtypes (BCSQ-12) into German and examined these burnout subtypes in the German working population. Using a non-probabilistic online quota sampling method, 616 employees were surveyed. The BCSQ-12 was translated and evaluated for its psychometric properties (EFA, Varimax rotation). Burnout subtypes were identified through hierarchical and K-means cluster analysis and analysed for symptomatologic, work-related and individual differences, particularly structural impairment (ANOVA). Additional questionnaires assessed burnout (MBI-GS-D), depression (PHQ-9), work engagement (UWES-9), job demands (COPSOQ), social support (MSPSS), and structural impairment (OPD-SQ). The BCSQ-12 demonstrated satisfactory psychometric properties and confirmed its three-factor structure. The cluster analyses revealed four profiles: non-burned-out, frenetic, underchallenged and worn-out, which differed in terms of the symptomatologic, work-related and individual factors. The pattern of resources and demands between the burnout subtypes indicates a deterioration from the frenetic to the underchallenged to the worn-out employee. The present results emphasize possible advantages of including burnout profiles into the general assessment of burnout and indicate the need for tailored interventions.

## Introduction

### Burnout: Conceptualization and diagnostic challenges

Since Freudenberger [1] described burnout as a phenomenon, it has become a core concept in health as well as occupational and organizational psychology [2]. To date, burnout is most commonly understood through the concept proposed by Maslach and Jackson [3], which views burnout as the reaction to chronic stress at work [3]. Burnout is characterized by the three key dimensions: emotional exhaustion, cynicism, and professional efficacy. Exhaustion describes the lack of energy, which can manifest itself both physically and psychologically, while cynicism reflects the detached and negative attitude towards one´s work and coworkers. The third dimension, professional efficacy involves an individual´s sense of competence and success at work [3]. Although burnout affects 4–7% of the working population [4], it is not mentioned in the DSM-5 [5]. In the ICD-11 [6] burnout (QD85) is more precisely defined but still not classified as a medical condition [6]. This is at least partly due to the missing consensus on the definition and trajectories of burnout among researchers [7–15].

### An alternative burnout approach: Farber´s burnout subtypes

While the missing consensus is a challenge in some regards, it can also be understood as an advantage that describes a lively theoretical and conceptual debate. As an example Farber [16] also challenged the traditional definition of burnout in terms of its unified, albeit three-dimensional, definition and its aetiology. He defined three burnout subtypes – the frenetic, the underchallenged and the worn-out burnout subtype – each differing in terms of their sources for the development of burnout [17–19]. This approach was formalized by Montero-Marin and García-Campayo [17], who defined the three burnout subgroups as follows:

The *frenetic* burnout subtype is characterized by high levels of work engagement, ambition, and over-taking of work tasks. Although frenetic individuals initially work effectively and actively seek solutions to work-related problems, their overcommitment to work, depletion of their energy reserves, and lack of ability to know their limits leads to exhaustion [17]. The *underchallenged* burnout subtype, however, describes phenomena of boredom and indifference and refers to individuals who lack work-related commitment and motivation to their professional tasks. The lack of challenges and personal development opportunities, as well as the associated boredom, lead to burnout [17]. Finally, the *worn-out* burnout subtype is characterized by a lack of work-related commitment, neglect of his or her tasks at work, and a tendency to resign. This burnout subtype experiences their employer´s organizational culture as rigid, unappreciative, and uncontrollable, causing them to burn out [17]. By that, Farber´s [16] typology provides a possibly more comprehensive definition of burnout than the one usually assessed by Maslach´s approach [3]. It allows the identification of specific characteristics for each person and favours the development of specific burnout prevention and intervention programs [20].

### The Burnout Clinical Subtype Questionnaire (BCSQ-12): Development, validation and cross-cultural applications

To capture Farber´s [16] burnout subgroups in the working population, the Burnout Clinical Subtype Questionnaire was developed. It is currently available in a long version (BCSQ-36: [17]) and a short version (BCSQ-12: [21]). Compared to the long version, the short version is more economical and has better psychometric properties [22]. So far, the 3-factor structure of the BCSQ-12 [21] has been investigated in Spanish university employees [21] and could be confirmed in two studies on Spanish primary health care professionals [23] and secondary school teachers [22] as well as in Brazilian healthcare professionals [24]. Furthermore, the dimensions of the BCSQ-12 [21] were shown to correlate with the burnout dimensions of the Maslach Burnout Inventory (MBI: 3) [25,26]. The frenetic burnout subtype was most negatively associated with exhaustion, while the underchallenged burnout subtype was most positively associated with cynicism [25,26]. The worn-out burnout subtype was most strongly negatively associated with professional efficacy [25,26]. To date, there is only a German translation of the student version of the BCSQ-12 (BCSQ-12-SS: 19) [26]. However, the general version of the BCSQ-12 [21] has not been translated into German and has not yet been validated in the German working population. As a result, the scope of research using the BCSQ-12 [21] and the cross-national comparison of corresponding results is still limited.

### Burnout and depression: Overlapping constructs or distinct phenomena

While examining burnout subtypes using Farber`s [16] typological approach provides a nuanced understanding of burnout, both research and intervention strategies require the investigation of symptomatologic, work-related and individual factors associated with burnout. For example, in addition to the discussion on the definition and trajectories of burnout, the study results on the burnout-depression overlap are still inconclusive [7,27–37]. In two recent meta-analysis, overlaps between burnout and depression, especially between emotional exhaustion and depression, were found [7,38]. However, these overlaps were found to be insufficient to assume an identical construct [7,38]. In addition, in some longitudinal studies burnout predicted the development of depression [29,39–41], and others found an inverse [42,43] or reciprocal effect [41,44]. Some studies, however, proved that burnout and depression can occur simultaneously and develop in tandem [28,45,46]. These findings rather suggest that burnout is an equivalent to depression. In some studies, using a person-oriented approach, it was found that depression is most prevalent in severe compared to mild burnout profiles [47–49]. In a study that examined the burnout subtypes according to Farber [16] these results could be confirmed [26]. Here, depressiveness was most prominent in the worn-out, followed by the underchallenged and the frenetic burnout subtype [26].

### The role of work engagement in burnout development

Equally important is the meaning of work engagement, a positive and fulfilling work-related state characterized by high energy and resilience (vigor), enthusiasm and pride (dedication), and deep focus, where one becomes fully immersed in tasks (absorption) [32]. Previously, work engagement has been defined as either an opposing or independent construct in relation to burnout [32,50]. However, recent studies suggest a dialectic relationship that can be explained by the interaction between job demands and resources [51–54]. Regarding the burnout subtypes according to Farber [16] work engagement represents a classification criterion for the different burnout subtypes [17,20]. While the frenetic burnout subtype has a high level of engagement, the underchallenged and the worn-out burnout subtype are characterized by a lack of work engagement [17,20,23,26].

### Job demands: A key driver of burnout

According to the job demand resource model (JD-R; 51), persistent overload and job demands are important factors influencing burnout [51,55,56]. Previous studies have demonstrated a strong link between increased job demands and emotional exhaustion [57,58], which in turn is associated with the frenetic burnout subtype [25,26]. With regard to Farber´s [16] burnout subtypes, it was shown that individuals who belonged to the frenetic burnout subgroup, worked more hours per week than the other two burnout subtypes [18,21,26].

### The impact of social support on burnout development

In addition to job demands, work-related resources also have an impact on the development of burnout [51,56,59]. In this respect, social support is a well-studied resource that can prevent the development of burnout [60–62]. A meta-analysis showed that lack of social support is most strongly associated with emotional exhaustion [62]. This result could not be confirmed in the meta-analysis by Kim et al. [60]. Here, the association of lack of social support with cynicism was strongest [60]. Regarding the burnout subtypes according to Farber [16] the presence of social support was positively related to the frenetic, but not to the other two burnout subtypes [63]. Another study also showed that social support decreased from the frenetic to the underchallenged to the worn-out burnout subtype [26].

### Structural impairments: A risk factor for burnout

Although burnout is seen as an organizational rather than an individual problem, individual characteristics also play a significant role in the development of burnout [51,64–66]. In addition to the BIG-5 personality traits [67,68], core self-evaluation, optimism, proactive personality, hardiness, Type A personality [68], emotional intelligence and self-efficacy [69] have been studied in relation to burnout. The burnout subtypes according to Farber [16] also differ in terms of personality characteristics [23,63]. While the frenetic burnout subtype copes with stressful situations by venting emotions or by problem focused coping (active coping strategy), the underchallenged burnout subtype uses cognitive avoidance, and the worn-out burnout subtype tends to disengage from stressors (passive coping) [63]. In addition, the frenetic burnout subtype was found to have higher levels of self-compassion than the other two burnout subtypes [23]. One personality-level factor that is widely studied in German-speaking countries is the personality structure according to the Operationalized Psychodynamic Diagnosis system [70–73]. Personality structure defines a broad range of inner-psychic regulatory mechanisms that enable individuals to adapt more or less flexible to external and internal stress [71,74]. According to this, adaption to stressful situations is more difficult if the personality structure is impaired [71,74]. So far, structural impairment has been predominantly studied as a vulnerability factor in relation to mental disorders [75–82]. Moreover, several studies showed that structural impairment increases with the severity of the mental disorder [80,81,83]. So far, there are only a few studies focusing on the relation between structural impairment and burnout [74,84,85]. To our knowledge, it is still unclear, however, to what extent the burnout subtypes according to Farber [16] differ in terms of their associations with structural impairment. Since individual resources decrease from the frenetic to the underchallenged to the worn-out burnout subtype [26], we assumed that structural impairment is most prominent in the worn-out burnout subtype.

### Identifying burnout subtypes in the German working population: A person-oriented approach

Although Farber’s [16] typological approach is a promising alternative for studying burnout, it is still unexplored how many burnout subtypes can be identified in the German working population and whether these are differentially related to depressiveness, work engagement, job demands, social support, and structural impairment. Since low to moderate correlations between the three burnout subtypes were found, it can be suggested that there might be different burnout groups that are intra-individually homogenous but inter-individually heterogenous [21,26]. The person-oriented approach is one way to identify typical burnout profiles. So far, burnout profiles have mainly been studied according to the traditional understanding of burnout [8,48,49,86–88]. Currently, there is only one study among students in which the burnout subtypes according to Farber [16] were examined using a person-oriented approach [26]. Here, 5 different burnout subtypes were identified (healthy engaged, frenetic, underchallenged, mildly worn out, severely worn out) which partly differ from the profiles described by Montero-Marin and García-Campayo [17,26]. To date, however, there is no study in the German working population that focuses on the profiles of the burnout subtypes and their differences in terms of the described symptomatologic, work-related and individual factors.

### Aims and scope of the study

Based on the theoretical and empirical findings, the present study pursued four main objectives: First, we wanted to validate the BCSQ-12 [21] for the German working population. To this end, we first examined the internal consistency and dimensionality of the scales of the BCSQ-12 [21] (reliability and factorial validity). Secondly, we investigated whether there are correlations between the scales of the BCSQ-12 [21] and the MBI-GS-D [3] as expected (convergent validity). Thirdly, we wanted to identify the three burnout subtypes according to Farber [16] using a person-oriented approach. Finally, we wanted to investigate whether they differ in terms of burnout symptoms, depressiveness, work engagement, job demands, social support and structural impairment.

## Materials and methods

### Study design

The study protocol received approval by the ethics committee of Charité Universitätsmedizin Berlin (ID: EA4/180/21). The study adhered to the principles of the Declaration of Helsinki. Before taking part in the study, all participants were informed about its purpose, data protection policies, the voluntary nature of their participation, and their right to withdraw at any time. Additionally, they provided written informed consent before participating. The data for the present study were obtained from a sample designed to be representative of the German working population, considering age, gender, and place of residence. The population update for the year 2022 based on the 2011 Census of the Federal Statistical Office was used to determine the corresponding quotas [89]. The study is a cross-sectional survey, which was conducted via the market research institute *Bilendi GmbH*. Data collection started on January 17, 2024, and was completed on January 23, 2024.To ensure the quality of the collected data, a Relative Speed Index (RSI) of less than 2.0 was set. This ensured that questionnaires that were completed too quickly could be excluded from further data processing [90]. Inclusion criteria required participants to provide informed consent to the study conditions (based on [91]), complete the entire questionnaire, and fall within the predefined age range of 20–65 years.

### Sample

After n = 56 people were excluded from the calculations due to a too high RSI (RSI > 2.0), the sample consisted of N = 616 people. In the sample, the previously defined quotas for gender, age and place of residence were roughly met. Table 1 provides a comparison between the achieved quotas in the present sample and those from the 2022 population update, which is based on the census of the Federal Statistical Office [89]. In the present sample, the age range was between 20 and 64 years (M = 45.9, SD = 12.4). The binary classification of gender showed an almost equally distributed in the sample (50.8% men, 49.2% women). Most of the subjects were married or in a registered partnership (48.1%) and had children (53.9%). In addition, the majority of respondents stated that they had not suffered from a mental illness in the last 6 months (80.7%). In terms of educational attainment, most participants indicated that they had received a high school diploma or an equivalent qualification for universities or university of applied sciences (53.7%). In addition, most respondents indicated that they had completed either an apprenticeship or vocational training in the dual system or another qualification at a vocational school or college. Finally, more than half of the study participants worked full-time (58.3%) and had a net household income of between € 3600 and € 5000 (25.5%). Table 2 provides a summary of the socio-demographic attributes of the study participants.

**Table 1:** Sample description.

		Frequency in the sample	Frequency in the sample in %	German working population in %	Difference
Age	20 to under 35 years	140	22.70%	30.35%	−7.65%
	35 to under 50 years	191	31.00%	31.51%	−0.51%
	50 to under 65 years	285	46.30%	38.14%	8.16%
Gender	male	303	49.20%	50.53%	−1.33%
	female	313	50.80%	49.47%	1.33%
Region	north	108	17.50%	18.04%	−0.54%
	east	113	18.34%	16.97%	−1.37%
	south	171	27.80%	29.69%	−1.89%
	west	224	36.40%	35.29%	1.11%

Note. N = 616; Source for the corresponding quotas for the German working population: Federal Statistic Office. (2023). Fortschreibung des Bevölkerungsstandes auf Grundlage des Zensus 2011 [Update of the population status based on the 2011 census]; classification of the regions: North (Bremen, Hamburg, Mecklenburg-Western Pomerania, Schleswig-Holstein, Lower Saxony), East (Berlin, Brandenburg, Saxony, Saxony-Anhalt, Thüringen), South (Baden-Württemberg, Bavaria), West (Hesse, North Rhine-Westphalia, Rhineland-Palatinate, Saarland).

**Table 2:** Sociodemographic characteristics of participants.

	N	%
Total sample size	616	100.0
Marital status		
Single, without partnership	141	22.9
Single with partnership	108	17.5
Married/civil partnership	296	48.1
Separated	9	1.5
Divorced	47	7.6
Widowed	15	2.4
Children		
Yes	332	53.9
No	284	46.1
Mental disorder in the last 6 months		
Yes	119	19.3
No	497	80.7
Level of education		
High school diploma, entrance qualification for universities or universities of applied sciences	331	53.7
Middle school certificate, intermediate school leaving certificate, polytechnical Secondary school with completion of the 10th grade	220	35.7
Secondary school certificate, elementary school certificate, polytechnical Secondary school with completion of 8th or 9th	55	8.9
Graduation after a maximum of 7 years of school attendance	5	0.8
No school leaving certificate	5	0.8
Level of professional qualification		
University of applied sciences degree	184	29.9
Master, technician or equivalent technical college qualification	70	11.4
Degree from a school of educators	9	1.5
Professional academy	2	0.3
Technical college in the GDR	11	1.8
Training at a school for health and social professions	32	5.2
Preparatory training for the intermediate civil service in public administration	14	2.3
Apprenticeship or vocational training in the dual system or another qualification at a vocational school or college	235	38.1
Apprenticeship or vocational internship, pre-vocational training year	10	1.6
No vocational qualification	49	8.0
Employment		
Full-time employed	359	58.3
Part-time employed	100	16.2
Marginally employed	15	2.4
Unemployed or registered as unemployed	22	3.6
Retired or in early retirement	45	7.3
Temporarily or permanently unable to work	24	3.9
Pupil, student, trainee, participant in further training programs	26	4.2
Housewife or househusband, looking after children or caring for people in need for help	27	2.8
Not employed for other reasons	8	1.3
Household net income (n = 613)	49	8.0
Under 900 €	24	3.9
900 € to under 1300 €	42	6.8
1300 € to under 1500 €	22	3.6
1500 € to under 2000 €	58	9.4
2000€ to under 2600 €	81	13.1
2600 € to under 3600 €	123	20.0
3600 € to under 5000 €	157	25.5
5000 € to under 18000 €	104	16.9
over 18000 €	2	0.3

Note. N = Sample size.

### Measurement instruments

#### BCSQ-12.

The short version of the Burnout Questionnaire for Clinical Subtypes (BCSQ-12: 21) is a previously published and psychometrically validated instrument, developed by Montero-Marín et al. [21] that is used to assess the burnout subtypes according to Farber [16]. It consists of a total of 12 items, which capture the dimensions “overload”, (“I overlook my own needs to fulfil work demands”) “lack of development” (“My work doesn´t offer me opportunities to develop my abilities”) and “neglect” (“When things don´t turn out as well as they should, I stop trying”) with 4 items each. The frenetic burnout subtype is represented by the dimension “overload”, the underchallenged burnout subtype by the dimension “lack of development” and the worn-out burnout subtype by the dimension “neglect”. The items are rated using a seven-point Likert-scale ranging from “totally disagree” (1), “strongly disagree” (2), “disagree” (3), “unsure” (4), “agree” (5), “strongly agree” (6) to “totally agree” (7). The results are presented as scalar values, which are derived from the average value of each dimension. In previous studies, the BCSQ-12 (21) showed good psychometric properties (α ≥ .80) [21,25]. Since there is no German version of the BCSQ-12 [21], the English version [21] was translated into German by the first author. This version was then translated back into English by a native English translator with very strong proficiency in German. The resulting translated English version was then compared with the original English version of the BCSQ-12 [21]. For the final German version of the questionnaire, any inconsistencies in the wording were discussed and the items were adapted accordingly. The original English version along with the final German version and the English back-translation can be found in the supplementary material (S1 Table). Apart from the BCSQ-12 [21], which was translated and validated for the present study, all other instruments were established instruments that have been psychometrically evaluated in previous research.

#### MBI-GS-D.

In addition, the German version of the MBI-General Survey (MBI-GS-D; 3), obtained from Mind Garden, Inc. [92], was used to assess burnout. The MBI-GS was originally developed by Maslach et al. [3]. The MBI-GS-D [3] comprises a total of 16 items. The dimensions “emotional exhaustion” (“I feel emotionally drained from my work”) and “cynicism” (“I doubt the significance of my work”) are measured with 5 items each. The dimension “professional efficiency” comprises 6 items (“In my opinion, I´m good at my job”). The items are answered on a seven-point Likert-scale ranging from “never” (0), “a few times a year or less” (1), “once a month or less” (2), “a few times a month” (3), “once a week” (4), “a few times a week” (5) to “every day” (6) (3). The MBI-GS-D (3) proved satisfactory to good psychometric properties. The reliabilities of the three MBI-GS-D (3) scales in the present study can be considered as good (ω = .90 – ω = .91).

#### PHQ-9.

Depressiveness was assessed using the German version of the Brief Patient Health Questionnaire Mood Scale (PHQ-9: [93]), developed by Kroenke et al. [94] and validated in German by Löwe et al. [93]. With a total of 9 items, the PHQ-9 [93] captures the 9 symptom domains of depression within the last two weeks according to the DSM-5 (5) (“Little interest or pleasure in your activities”). The items are measured using a four-point Likert-scale ranging from “not at all” (0), “on single days” (1), “on more than half of the days” (2) to “almost every day” (3). The total score of the PHQ-9 [93] indicates the degree of depression. A score ≥ 10 indicates major depression, which can be divided into moderate (score range 10–14), marked (score range 15–19 points) and severe episodes (score range 20–27). A score of 5–10 indicates a mild depressive disorder and less than 5 points indicates no depressive disorder. For the PHQ-9 [93] good psychometric properties have been reported [95]. In the present study, the internal consistency of the PHQ-9 total score (ω = .91) can be considered as good.

#### UWES-9.

Work engagement was measured with the German short version of the Utrecht Work Engagement Scale (UWES-9: 96), originally developed by Schaufeli et al. [32] and validated in German by Sautier et al. [96]. This comprises a total of 9 items. The dimension “vitality” (“At my work, I feel that I´m bursting with energy”), “dedication” (“I am enthusiastic about my job”) and “absorption” (“When I am working, I forget everything else around me”) are measured with 3 items each. For the UWES-9 [96] a total score is recommended [96]. The items are answered on a seven-point Likert-scale ranging from “never” (0), “almost never” (1), “rarely” (2), “sometimes” (3), “often (4), “very often” (5) to “always” (6). For the UWES-9 [96] good psychometric properties have been reported [96]. In the present study, the internal consistency of the total score was ω = .96, which can be interpreted as good.

#### COPSOQ.

Job demands were measured using the “quantitative job demands” subscale of the Copenhagen Psychosocial Questionnaire (COPSOQ: [97]), developed by Kristensen and Borg [98] and adapted into German by Nübling et al. [97]. With a total of 7 items this subscale captures whether it is possible to complete all tasks in time without falling behind or having to work overtime (“How often does it happen that you do not have enough time to complete all of your tasks?). The items are recorded on a five-point Likert-scale ranging from “always” (4), “often” (3), “sometimes” (2), “rarely” (1) to “never/almost never” (0). The internal consistency of the subscale in the study by Nübling et al. [97] was α = 0.82, which can be considered good. In the present study, the internal consistency was ω = .71. This can be interpreted as acceptable**.**

#### MSPSS.

Social support was measured using the German version of the Multidimensional Scale of Perceived Social Support (MSPSS: 99), originally developed by Zimet et al. [99]. The German version was provided directly by one of the authors of the original version (Gregory D. Zimet) upon request. With a total of 12 items and 3 subscales, this scale captures social support from significant others (“There is a special person who is around when I am in need”), family (“My family really tries to help me”) and friends (“I can count on my friends when things go wrong”) with 4 items each. The items were rated on a seven-point Likert-scale from “very strongly disagree” (1), “strongly disagree” (2), “mildly disagree (3), “neutral (4), “mildly agree” (5), strongly agree (6) and very strongly agree (7). The psychometric criteria for the MSPSS [99] were reported to be satisfactory to very good [100]. With a ω -value of.95 in the present study, the internal consistency can be considered as good.

#### OPD-SQ.

Structural impairment according to the Operationalized Psychodynamic Diagnosis system [71] was measured using the OPD-Structure Questionnaire (OPD-SQ: 101), developed by Ehrenthal et al [101]. The OPD-SQ [101] comprises a total of 95 items that capture the 4 dimensions of perception (“It is difficult for me to describe myself”, regulation (“I have repeatedly been told that I do not pay enough attention to the needs of others”), emotional communication (“It is often unclear to me what I am feeling”) and attachment (“It is difficult for me to ask others for help”) in relation to the self and others [101]. Each main scale is composed of 2–3 subscales, which in turn consist of 3–7 items each. Table 3 provides an overview of the dimensions and subscales of structural impairment [71]. The items are recorded on a five-point Likert-scale that can be rated from “strongly disagree” (0), “disagree” (1), “neither agree nor disagree” (2), “agree” (3) to “strongly agree” (4). Higher scores indicate greater structural impairment. The OPD-SQ [101] has satisfactory to good psychometric properties [101–103]. In the present study the reliability of the OPD-SQ [101] total score was ω = .98, which can be rated as good. The reliabilities of the eight OPD-SQ [101] scales in the present study can be considered as acceptable to good (ω = .79 – ω = .94).

**Table 3:** The dimensions and subscales of the OPD-SQ.

Self-perception	Object-perception
Reflecting oneself	Differentiating self from others
Differentiating affects	Perceiving others holistically
Having a sense of identity	Perceiving others realistically
Self-regulation	Regulation of Relationships
Controlling impulses	Protecting relationships
Tolerating affects	Balancing interests
Regulating one´s self-worth	Anticipating the reaction of others
Internal emotional communication	External emotional communication
Experiencing affects	Establishing emotional contact
Using fantasies	Communicating affects
Experiencing the bodily self	Being empathetic
Attachment to internal objects	Attachment to external objects
Internalizing positive object representations	Attaching to others
Using positive introjects	Accepting help
Forming variable attachments	Detaching from relationships

Source. OPD Task Force [71].

### Data analysis

Statistical analyses were conducted using SPSS (version 27). First, the descriptive statistics were calculated, which included the means, standard deviations, skewness, kurtosis, and minimum and maximum values of the BCSQ-12 [21]. McDonalds ω [104] was calculated to determine internal consistency. In accordance with Cronbach’s alpha, these effects were interpreted as acceptable (ω = .7) or good (ω = .8) [105].

As the BCSQ-12 [21] has mainly been validated in Spanish- [21–23] or Portuguese- [24] speaking samples, evidence for structural invariance of the newly translated version in the German working population is lacking. To account for potential cultural and linguistic differences in the factor structure and to assess its applicability in the German work context, an exploratory approach was deemed appropriate.

The principal axis method with varimax rotation was applied, and the number of factors was determined using the Kaiser criterion (eigenvalues > 1) [106] as well as the Cattel´s scree-test [107]. To investigate the convergent validity of the BCSQ-12, Pearson Correlations with the dimensions of the MBI-GS-D [3] were also calculated. According to Cohen [108], these effects were interpreted as small (r ≥ .1), medium (r ≥ .3) and large (r ≥ .5).

In order to investigate whether the three Montero-Marin-Burnout subtypes can be distinguished in the German working population, a two-stage explorative cluster analysis procedure according to Bergman and Magnusson [109] was carried out. Before conducting the cluster analyses, the three subscales of the BCSQ-12 were first z-standardized. In a first step, a hierarchical agglomerative cluster analysis was carried out, using the squared Euclidean distance as a measure of similarity and the Ward method to minimize differences within the clusters. The optimal number of clusters was selected using the dendrogram. In a second step, a K-means cluster analysis was performed to determine the final clusters. No further tests were conducted to assess the stability of the cluster solution.

After identifying the burnout subtypes using cluster analysis, the differences between the burnout subtypes in terms of burnout symptoms, depression, work engagement, job demands, social support and structural impairment were analysed using analyses of variance (ANOVAS). In addition, post-hoc tests were carried out to determine which groups differed significantly from one another. For homogeneous variances, the post-hoc comparisons were carried out using the Bonferroni test and the Games Howell test for inhomogeneous variances tests. The partial Eta squared (ηp2) was calculated to evaluate the effect size. According to Cohen [108], the effect sizes were interpreted as small (0.01 ≤ ηp2 < 0.06), medium (0.06 ≤ ηp2 < 0.14) or large (ηp2 ≥ 0.14) effects.

## Results

### Reliability, factor validity, and convergent validity

Table 4 provides an overview of the descriptive statistics of the metric variables in the present study. Regarding the reliability, the internal consistencies of the BCSQ-12 scales were tested. In this respect, all three scales of the BCSQ-12 showed good results (see Table 5). In addition, an exploratory factor analysis was carried out to check the factorial validity. To test the suitability of the factor model, the Kaiser-Meyer-Olkin test and the Bartlett test for sphericity were performed. In the present study, the KMO value was.90 and the Bartlett’s test for sphericity was significant (Χ^2^ = 4449.65, *df* = 66, < .001), thus fulfilling the requirements for an exploratory factor analysis. The EFA resulted in a 3-factor solution which required no forcing. The three components fulfilled the Kaiser criterion [106] (eigenvalues > 1) and Cattell’s scree-plot test [107]. In addition, the three components explained 64.32% of the total variance. The first factor, overload, showed an eigenvalue of λ_1_ = 5.94 and explained 46.69% of the variance. The second factor, lack of development, showed an eigenvalue of λ_2_ = 1.60 and explained 10.37% of the variance. The third factor, neglect, showed an eigenvalue of λ_3_ = 1.16 and explained 7.27% of the variance. Table 6 shows the factor loading matrix for the three-factor solution with 12 items as well as the means, the standard deviations and the commonalities. Regarding convergent validity, correlations were found between the “overload” and “exhaustion” (r = .57) and between the “lack of development” and “cynicism” (r = .73). Although the expected negative correlation was found between “neglect” and “professional efficacy” (r = −.30), the correlations between the “neglect” and “cynicism” scales were higher (r = .60). Table 7 provides an overview of the intercorrelations between the scales of the BCSQ12 and the MBI-GS-D [3].

**Table 4:** Descriptive statistics.

	M	SD	Skew	Kurtosis	Min	Max
Burnout (BCSQ-12)						
Overload	3.37	1.56	0.21	−0.73	1.00	7.00
Lack of development	3.32	1.62	0.22	−0.86	1.00	7.00
Neglect	2.69	1.35	0.68	0.02	1.00	7.00
Burnout (MBI-GS-D)						
Exhaustion	2.63	1.67	0.19	−0.94	0.00	6.00
Cynicism	2.08	1.78	0.59	−0.79	0.00	6.00
Professional efficacy	4.45	1.42	−1.46	2.10	0.00	6.00
Depressiveness	0.77	0.67	1.07	0.67	0.00	3.00
Work engagement	3.40	1.47	−0.54	−0.19	0.00	6.00
Job demands	1.65	0.79	0.31	−0.06	0.00	4.00
Social support	5.36	1.38	−1.04	0.72	1.00	5.00
Structural impairment	1.46	0.66	0.31	−0.32	0.15	3.60
Self-perception	1.17	0.89	0.62	−0.25	0.00	4.00
Other-perception	1.53	0.69	0.13	−0.27	0.00	3.76
Self-regulation	1.31	0.78	0.45	−0.36	0.00	3.85
Regulation of relationships	1.39	0.75	0.27	−0.25	0.00	4.00
Internal emotional communication	1.28	0.66	0.54	0.18	0.00	3.82
External emotional communication	1.62	0.68	0.28	−0.14	0.00	4.00
Attachment to internal object	1.64	0.87	0.19	−0.55	0.00	4.00
Attachment to external object	1.89	0.79	0.10	−0.39	0.00	4.00

Note: N = 616, M = Mean, SD = standard deviation.

**Table 5:** Internal consistency of the BCSQ-12 scales.

Scale	McDonald´s ω
Overload	.88
Lack of development	.88
Neglect	.86

**Table 6:** Results of an exploratory factor analysis of the German BCSQ-12.

BCSQ-12	Overload	Lack of development	Neglect	M	SD	h^2^
Item 7	**.80**	.17	.26	3.00	1.85	.74
Item 4	**.74**	.10	.14	3.18	1.78	.58
Item 10	**.74**	.27	.21	3.35	1.81	.66
Item 1	**.71**	.28	.15	4.00	1.87	.60
Item 8	.20	**.86**	.24	3.40	2.04	.84
Item 2	.22	**.84**	.23	3.51	2.00	.81
Item 5	.35	**.63**	.40	2.90	1.73	.67
Item 11	.17	**.48**	.28	3.46	1.91	.34
Item 6	.18	.17	**.85**	2.42	1.49	.78
Item 3	.16	.34	**.70**	2.93	1.63	.63
Item 9	.18	.22	**.67**	2.50	1.67	.53
Item 12	.28	.33	**.60**	2.89	1.67	.55

Note. N = 616. The extraction method was principal axis procedure with varimax rotation.

M = mean, SD = standard deviation, h^2^ = communalities.

**Table 7:** Intercorrelations between the BCSQ-12 and the MBI-GS-D.

Variable	1	2	3	4	5	6
1. Overload	1					
2. Lack of development	.52**	1	.			
3. Neglect	.47**	.61**	1	.		
4. Exhaustion	.57**	.60**	.53**	1		
5. Cynicism	.41**	.73**	.60**	.70**	1	
6. Professional efficacy	.02	−.16**	−.30**	−.04**	−.21**	1

Note. N = 616.

* p < .05, ** p < 01, corrected p-value (Bonferroni) *** p < .001.

### Burnout profiles in the German working population

**Fig 1:**
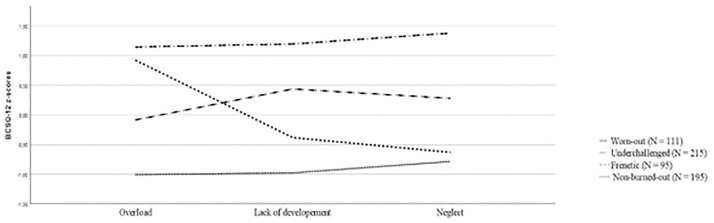
Burnout profiles based on the BCSQ-12 z-scores.

The cluster analysis with the three BCSQ-12 subscales revealed two to five burnout subtypes in the present sample. We considered the 4-cluster solution (non-burned-out employees, frenetic employees, underchallenged employees and worn-out employees) to be the appropriate structure, as important information was missing in the 2- and 3- cluster solutions. In the 5-cluster solution, a further cluster of people emerged which was like another group in terms of burnout symptoms but had a lower level of severity and therefore did not provide any further conceptual clarity to describe the group**.** The BCSQ-12 z-scores were used to describe the four profiles. The four profiles were labelled as follows: *non-burned-out employees, frenetic employees, underchallenged employees* and *worn-out employees.* The *non-burned-out employees* (31.66%) showed no burnout symptoms and had low scores on all three BCSQ-12-subscales. The following 3 burnout subtypes corresponded to Montero-Marin’s burnout typology [21]. The *frenetic employees* (15.42%) showed high scores for overload as well as low scores for lack of development and neglect. The *underchallenged employees* (34.90%), who represented the largest group, had moderate scores for lack of development and neglect as well as slightly negative scores for overload. Finally, the *worn-out employees* (18.02%) had the highest scores for neglect as well as high scores for lack of development and overload. Fig 1 shows the four burnout profiles based on the BCSQ-12 z-scores.

### Burnout profiles and their differences in symptomatologic, work-related, and individual factors

Table 8 shows how the burnout profiles differ in terms of burnout symptoms, depressiveness, work engagement, job demands, social support and structural impairment.

**Table 8:** Burnout profiles and their differences in burnout symptoms, depression, work engagement, job demands, social support, and structural impairment.

	non-burned-out	frenetic	underchallenged	worn-out	F(3,612)	ηp^2^	Post-hoc analysis
	M (SD)	M (SD)	M (SD)	M (SD)			
BCSQ-12							
Overload	1.81 (0.76)	4.81 (0.94)	3.25 (0.88)	5.16 (0.95)	461.57***	0.69	
Lack of development	1.73 (0.81)	2.70 (1.14)	4.03 (1.02)	5.26 (0.93)	380.90***	0.65	
Neglect	1.63 (0.70)	1.83 (0.74)	3.10 (0.77)	4.54 (1.12)	345.84***	0.63	
MBI-GS-D							
Exhaustion	1.36 (1.14)	2.64 (1.50)	2.85 (1.34)	4.43 (1.27)	135.73***	0.40	1 < 2, 3, 4; 2, 3 < 4^a^
Cynicism	0.77 (0.95)	1.29 (1.23)	2.58 (1.59)	4.07 (1.36)	169.61***	0.45	1 < 2 < 3 < 4^a^
Professional efficacy	4.68 (1.67)	5.12 (0.82)	4.22 (1.30)	3.95 (1.26)	16.08***	0.07	1 < 2, 3, 4; 2 > 3, 4^a^
Depressiveness	0.37 (0.44)	0.75 (0.66)	0.81 (0.53)	1.39 (0.74)	74.98***	0.27	1 < 2, 3, 4; 2, 3 < 4^a^
Work engagement	3.91 (1.58)	4.34 (0.97)	3.00 (1.20)	2.50 (1.33)	49.80***	0.20	1 < 2 > 3 > 4^a^
Job demands	1.22 (0.62)	1.99 (0.74)	1.60 (0.72)	2.26 (0.77)	60.16***	0.23	1 < 2, 3, 4; 2 > 3 < 4^b^
Social support	5.91 (1.21)	5.41 (1.33)	5.19 (1.29)	4.70 (1.52)	22.00***	0.10	1 > 2, 3, 4; 2, 3 > 4^a^;
Structural impairment	1.10 (0.58)	1.48 (0.69)	1.55 (0.54)	1.90 (0.69)	44.18***	0.19	1 < 2, 3, 4; 2, 3 < 4^a^
Self-perception	0.75 (0.71)	1.16 (0.97)	1.25 (0.77)	1.75 (0.97)	36.27***	0.15	1 < 2, 3, 4; 2, 3 < 4^a^
Object perception	1.19 (0.66)	1.55 (0.64)	1.62 (0.57)	1.92 (0.74)	32.76***	0.14	1 < 2, 3, 4; 2, 3 < 4^a^
Self-regulation	0.92 (0.67)	1.32 (0.83)	1.42 (0.66)	1.78 (0.79)	36.83***	0.15	1 < 2, 3, 4; 2, 3 < 4^a^
Regulation of relationships	1.00 (0.68)	1.42 (0.77)	1.52 (0.62)	1.77 (0.79)	34.02***	0.14	1 < 2, 3, 4; 2, 3 < 4^a^
Internal emotional communication	1.00 (0.55)	1.23 (0.73)	1.35 (0.58)	1.69 (0.70)	30.57***	0.13	1 < 2, 3, 4; 2, 3 < 4^a^
External emotional communication	1.31 (0.60)	1.60 (0.72)	1.71 (0.57)	2.03 (0.72)	32.88***	0.14	1 < 2, 3, 4; 2, 3 < 4^a^
Attachment to internal objects	1.17 (0.81)	1.80 (0.85)	1.73 (0.72)	2.17 (0.90)	40.54***	0.17	1 < 2, 3, 4; 2, 3 < 4^b^
Attachment to external objects	1.59 (0.75)	1.98 (0.84)	1.93 (0.67)	2.25 (0.73)	20.83***	0.10	1 < 2, 3, 4; 3 < 4^b^

Note. N = 616, ηp^2^ = partial Eta-squared, M = mean, SD = standard deviation, 1 = non-burned-out, 2 = frenetic, 3 = underchallenged, 4 = worn-out. a = Games-Howell post-hoc analysis; b = Bonferroni post-hoc analysis.

* p < .05, ** p < 01, *** p < .001.

#### Burnout profiles and their differences in burnout symptoms.

The four burnout profiles differed statistically significantly with regard to the three burnout symptoms exhaustion (F(3,612) = 135.73, p < .001), cynicism (F(3,612) = 294.02, p < .001) and professional efficacy (F(3,612) = 16.08, p < .001).

The non-burned-out employees showed the lowest exhaustion scores, followed by the frenetic employees, the underchallenged employees and the worn-out employees. The post-hoc analysis revealed significant differences between the non-burned-out employees compared to the frenetic employees (p < .001, MDiff = −1.28, 95%-CI [−1.74; −0. 83]), underchallenged employees (p < .001, MDiff = −1.49, 95%-CI [−1.80; −1.17]) and worn-out employees (p < .001, MDiff = −3.07., 95%-CI [−3.45; −2.69]) in terms of exhaustion. In addition, there were statistically significant differences between the frenetic employees and the worn-out employees (p < .001, MDiff = −1.79, 95%-CI [−2.30; −1.28]) and between the underchallenged employees and the worn-out employees (p < .001, MDiff = −1.58., 95%-CI [−1.98; −1.19]) with regard to exhaustion. Although the frenetic employees had lower exhaustion scores compared to the underchallenged employees, this difference was not statistically significant (p = .67, MDiff = −0.20., 95%-CI [−0.67; 0.26]).

Furthermore, the non-burned-out employees had the lowest cynicism scores, followed by the frenetic employees, the underchallenged employees and the worn-out employees. The post-hoc analysis revealed significant differences between the non-burned-out employees and the frenetic employees (p = .002, MDiff = −0.52., 95%-CI [−0.89; −0. 15]), the underchallenged employees (p < .001, MDiff = −1.80, 95%-CI [−2.14; −1.47]) and the worn-out employees (p < .001, MDiff = −3.29, 95%-CI [−3.67; −2.91]) in terms of cynicism. Additionally, there were statistically significant differences between the frenetic employees and the underchallenged employees (p < .001, MDiff = −1.29, 95%-CI [−1.72; −0.86]) and between the frenetic employees and the worn-out employees (p < .001, MDiff = −2.77, 95%-CI [−3.24; −2.31]) with regard to cynicism. In this respect, there was also a statistically significant difference between the underchallenged employees and the worn-out employees (p < .001, MDiff = −1.49, 95%-CI [−1.92; −1.05]).

In addition, the frenetic employees showed the highest professional efficacy, followed by the non-burned-out employees, the underchallenged employees and the worn-out employees. The post-hoc analysis revealed significant differences between the non-burned-out employees and the frenetic employees (p = .02, MDiff = −0.44, 95%-CI [−0.82; −0. 06]), the underchallenged employees (p = .01, MDiff = 0.46, 95%-CI [0.07; 0.85]) and the worn-out employees (p < .001, MDiff = 0.72, 95%-CI [0.28; 1.16]) in terms of professional efficacy. There were also statistically significant differences between the frenetic employees and the underchallenged employees (p < .001, MDiff = 0.90, 95%-CI [0.58; 1.21]) and between the frenetic employees and the worn-out employees (p < .001, MDiff = 1.16., 95%-CI [0.79; 1.54]) in terms of professional efficacy. Although the underchallenged employees showed higher professional efficacy compared to the worn-out employees, this difference was not statistically significant (p = .28, MDiff = 0.27, 95%-CI [−0.12; 0.65]).

#### Burnout profiles and their differences in depressiveness.

The four burnout profiles also differed in terms of depression (F(3,612) = 74.98, p < .001). The non-burned-out employees had the lowest depression scores, followed by the frenetic employees, the underchallenged employees and the worn-out employees. The post-hoc analysis revealed significant differences between the non-burned-out employees and the frenetic employees (p < .001, MDiff = −0.37, 95%-CI [−0.57; −0.18]), the underchallenged employees (p < .001, MDiff = −0.43, 95%-CI [−0.56; −0.31]) and the worn-out employees (p < .001, MDiff = −1.01, 95%-CI [−1.21; −0.82]) in terms of depression. In addition, there were statistically significant differences between the frenetic employees and the worn-out employees (p < .001, MDiff = −0.64, 95%-CI [−0.89; −0.39]) and between the underchallenged employees and the worn-out employees (p < .001, MDiff = −0.58, 95%-CI [−0.79; −0.38]) with regard to depression. Although the frenetic employees had lower depression scores than the underchallenged employees, this difference was not statistically significant (p = .87, MDiff = −0.06, 95%-CI [- 0.26; 0.14]).

#### Burnout profiles and their differences in work engagement.

Additionally, the four burnout profiles differed in terms of work engagement (F(3,612) = 49.80, p < .001). The frenetic employees showed the highest work engagement, followed by the non-burned-out employees, the underchallenged employees and the worn-out employees with the lowest work engagement. The post-hoc analysis revealed statistically significant differences between the non-burned-out employees and the frenetic employees (p = .02, MDiff = −0.43., 95%-CI [−0.82; −0. 04]), the underchallenged employees (p < .001, MDiff = 0.92, 95%-CI [0.56; 1.28]) and the worn-out employees (p < .001, MDiff = 1.41, 95%-CI [0.97; 1.85]) in terms of work engagement. Moreover, there were statistically significant differences between the frenetic employees and the underchallenged employees (p < .001, MDiff = 1.35, 95%-CI [1.02; 1.68]) and between the frenetic employees and the worn-out employees (p < .001, MDiff = 1.84, 95%-CI [1.42; 2.26]) with regard to work engagement. There was also a statistically significant difference between the underchallenged employees and the worn-out employees (p = .01, MDiff = 0.49, 95%-CI [0.10; 0.88]) in terms of work engagement.

#### Burnout profiles and their differences in job demands.

The four burnout profiles also differed in terms of job demands (F(3,612) = 60.16, p < .001). The non-burned-out employees showed the lowest job demands, followed by the underchallenged employees, the frenetic employees and the worn-out employees. The post-hoc analysis showed statistically significant differences between the non-burned-out employees and the frenetic employees (p < .001, MDiff = −0.77, 95%-CI [−1.01; −0.54]), the underchallenged employees (p < .001, MDiff = −0.38, 95%-CI [−0.57; −0.20]) and the worn-out employees (p < .001, MDiff = −1.04, 95%-CI [−1.26; −0.82]) in terms of job demands. In addition, there were statistically significant differences between the frenetic employees and the underchallenged employees (p < .001, MDiff = 0.39; 95%-CI [0.16; 0.62]) and between the underchallenged employees and the worn-out employees (p < .001, MDiff = −0.65, 95%-CI [−0.87; −0.44]) regarding job demands. Although the frenetic employees reported higher job demands compared to the worn-out employees, this difference was not statistically significant (p = .05, MDiff = 0.26, 95%-CI [−0.52; −0.00]).

#### Burnout profiles and their differences in social support.

Besides, the four burnout profiles differed in terms of social support (F(3,612) = 22.00, p < .001). The non-burned-out employees showed the highest social support, followed by the frenetic employees, the underchallenged employees and the worn-out employees with the lowest social support. The post-hoc analysis revealed statistically significant differences between the non-burned-out employees and the frenetic employees (p = .01, MDiff = 0.50, 95%-CI [0.08; 0.92]), the underchallenged employees (p < .001, MDiff = 0.72, 95%-CI [0.40; 1.04]) and the worn-out employees (p < .001, MDiff = 1.21, 95%-CI [0.78; 1.65]) in terms of social support. There were also statistically significant differences between the frenetic employees and the worn-out employees (p < .01, MDiff = 0.71, 95%-CI [0.20; 1.23]) with regard to social support. There were also statistically significant differences between the underchallenged employees and the worn-out employees (p = .02, MDiff = 0.49, 95%-CI [0.05; 0.93]) with regard to social support. Although the frenetic employees showed a higher level of social support compared to the underchallenged employees, this difference was not statistically significant (p = .52, MDiff = 0.22, 95%-CI [−0.20; 0.64]).

#### Burnout profiles and their differences in structural impairment.

Finally, there were statistically significant differences between the four burnout profiles in terms of structural impairment (F(3,612) = 44.18, p < .001) and its facets such as self-perception (F(3,612) = 36.27, p < .001), object perception (F(3,612) = 32.76, p < .001), self-regulation (F(3,612) = 36. 83, p < .001), relationship regulation (F(3,612) = 34.02, p < .001), internal emotional communication (F(3,612) = 30.57, p < .001), external emotional communication (F(3,612) = 32.88, p < .001), attachment to internal objects (F(3,612) = 40.54, p < .001) and attachment to external objects (F(3,612) = 20.83, p < .001). An overview of the post hoc analyses between the burnout subtypes, the structural impairment and its facets can be found in Table 9. The non-burned-out employees showed the lowest scores in terms of structural impairment and its facets, self-perception, object-perception, self-regulation, regulation of relationships, internal emotional communication and external emotional communication, followed by the frenetic employees, the underchallenged employees and the worn-out employees. In addition, the non-burned-out employees showed the lowest scores in terms of attachment to internal objects and attachment of external objects, followed by the underchallenged employees, the frenetic employees and the worn-out employees.

**Table 9:** Burnout profiles and their differences in structural impairment and it´s facets.

	Burnout subtype		p	MDiff	95%-CI		Burnout subtype		p	MDiff	95%-CI
Structural impairment											
	1.	2.	<.001***	−0.38	[-0.59; -0.17]						
		3.	<.001***	−0.45	[-0.59; -0.31]						
		4.	<.001***	−0.80	[-1.00; -0.60]						
	2.	3.	.81	−0.07	[-0.28; -0.14]						
		4.	<.001***	−0.42	[-0.67; -0.17]						
	3.	4.	<.001***	−0.35	[-0.54; -0.15]						
Self-perception						Object-perception					
	1.	2.	.002**	−0.41	[-0.71; - 0.12]		1.	2.	<.001***	−0.36	[-0.57; -0.15]
		3.	<.001***	−0.51	[-0.70; -0.32]			3.	<.001***	−0.43	[-0.58; -0.27]
		4.	<.001***	−1.01	[-1.28; -0.73]			4.	<.001***	−0.73	[-0.94; -0.51]
	2.	3.	.84	−0.09	[-0.39; 0.20]		2.	3.	.82	−0.07	[0.26; 0.13]
		4.	<.001***	−0.59	[-0.94; -0.24]			4.	<.001***	−0.37	[0.62; -0.12]
	3.	4.	<.001***	−0.50	[-0.77; - 0.22]		3.	4.	.002**	−0.30	[-0.51; -0.09]
Self-regulation						Regulation of realtionships					
	1.	2.	<.001***	−0.40	[-0.65; -0.14]		1.	2.	<.001***	−0.42	[-0.66; -0.18]
		3.	<.001***	−0.50	[-0.67; -0.33]			3.	<.001***	−0.52	[-0.69; -0.36]
		4.	<.001***	−0.86	[-1.09; -0.63]			4.	<.001***	−0.77	[-1.00; -0.54]
	2.	3.	.70	−0.11	[-0.36; 0.15]		2.	3.	.64	−0.11	[-0.34; 0.13]
		4.	<.001***	−0.47	[-0.76; -0.17]			4.	.01*	−0.35	[-0.63; -0.07]
	3.	4.	<.001***	−0.36	[-0.59; -0.13]		3.	4.	.03	−0.25	[-0.47; -0.02]
Internal emotional communication						External emotional communication					
	1.	2.	.03	−0.24	[-0.46; -0.02]		1.	2.	.01*	−0.29	[-0.51; -0.07]
		3.	<.001***	−0.35	[-0.50; -0.21]			3.	<.001***	−0.40	[-0.55; -0.25]
		4.	<.001***	−0.69	[-0.89; -0.49]			4.	<.001***	−0.73	[-0.94; -0.52]
	2.	3.	.52	−0.12	[-0.34; 0.10]		2.	3.	.58	−0.11	[-0.32; 0.11]
		4.	<.001***	−0.45	[-0.71; -0.19]			4.	<.001***	−0.44	[-0.70; -0.18]
	3.	4.	<.001***	−0.33	[-0.53; -0.14]		3.	4.	<.001***	−0.33	[-0.54; -0.13]
Attachment to internal objects						Attachment to external objects					
	1.	2.	<.001***	−0.63	[-0.89; -0.36]		1.	2.	<.001***	−0.40	[-0.64; -0.15]
		3.	<.001***	−0.56	[-0.77; -0.35]			3.	<.001***	−0.35	[-0.54; -0.15]
		4.	<.001***	−1.00	[-1.25; -0.75]			4.	<.001***	−0.67	[-0.90; -0.44]
	2.	3.	1.00	0.07	[-0.19; 0.33]		2.	3.	1.00	0.05	[-0.19; 0.29]
		4.	.01*	−0.37	[-0.66; -0.07]			4.	.05	−0.27	[-0.55; - 0.00]
	3.	4.	<.001***	−0.44	[-0.69; -0.19]		3.	4.	.001**	−0.32	[-0.55; -0.09]

Note. Source: OPD Task Force, 2011, 2023; MDiff = Difference in mean value, 95%-CI = 95%-Confidence interval, 1 = Non-burned-out employees, 2 = Frenetic employees, 3 = Underchallenged employees, 4 = Worn-out employees.

* p < .05, ** p < .01, *** p < .001.

## Discussion

### Study objectives

The objective of the current study was to translate the BCSQ-12 [21] into German investigate the psychometric properties of the BCSQ-12 and its correlations with burnout symptoms according to the MBI-GS-D [3] in a representative sample of the German working population. In addition, four different burnout profiles were identified and examined for their differences in burnout symptoms, depression, work engagement, job demands, social support and structural impairment.

### Factor structure and validation of the BCSQ-12

The exploratory factor analysis revealed three factors, thus replicating the three-factor structure of the BCSQ-12 [21,22]. In addition, the items of the BCSQ-12 have both high factor loadings and high communalities [110,111]. Only item 11 showed low commonality, so the quality of this item should be improved in future studies. Furthermore, all three scales of the BCSQ-12 showed good internal consistencies. Finally, medium to large intercorrelations were found between the three scales of the BCSQ-12. This is in line with the previous study results [25,26] and indicates an overlap between the three burnout subtypes. Regarding convergent validity, the expected correlations between “overload” and “exhaustion”, “lack of development” and “cynicism” and between “neglect” and “professional efficacy” were found. Contrary to expectations, the subscale “neglect” of the BCSQ-12 correlated higher with the subscale “cynicism” than with the subscale “professional efficacy” scale of the MBI-GS-D [3]. However, this finding is in line with the study results by Demarzo et al. [24].

### Identification and characteristics of burnout subtypes

In order to investigate whether the three burnout subtypes can be found in the German working population, a two-stage cluster analysis procedure [109] was carried out. In addition to non-burned-out employees, the three burnout subtypes according to Farber [16] were identified in the present sample: The *non-burned-out employees* did not report any burnout symptoms and had the most favourable profile overall. In previous studies investigating burnout using a person-centred approach, profiles with non-burned-out subjects could also be identified [112–117].

The *non-burned-out employees* in the present study were the least depressed, the most engaged, had the least job demands and the most social support. The study by Bauernhofer et al. [26] also found non-burned-out study participants with a similarly favourable profile. In addition, the non-burned-out employees of the present study had the least structural impairments.

The *frenetic employees* stated that they mainly overloaded themselves. In addition, they showed a higher level of professional efficiency and work engagement compared to the non-burned-out employees, the underchallenged employees and the worn-out employees. The frenetic employees thus correspond to the burnout subtype profile of Montero-Marin and García-Campayo [17]. However, the frenetic employees were also more exhausted and cynical than the non-burned-out employees. This is consistent with previous studies that identified groups of engaged individuals who also showed signs of exhaustion [54,118,119]. It was also found that these people are quite successful in the short term, but have a greater fear of failure, an increased experience of stress and an increased risk of developing depression [119]. Moreover, the frenetic employees had a higher level of job demands compared to the non-burned-out employees and the underchallenged employees but also a higher level of social support compared to the underchallenged employees and the worn-out employees. The latter results are consistent with previous findings in which the frenetic burnout subtype showed higher levels of social support than the other burnout subtypes [26,63]. Moreover, previous studies showed that social support is associated with higher work engagement [120,121] and lower burnout symptoms [60,62]. Possibly, social support served the frenetic employees in the present study as a resource to achieve a higher level of work engagement and to cope with higher job demands. Overall, frenetic employees seem to be more likely to use active coping strategies, which is reflected in their higher professional efficacy and their increased work engagement This is in line with previous findings in which the frenetic burnout subtype was associated with problem-oriented coping strategies [63]. Finally, the frenetic employees showed more structural impairments than the non-burned-out employees and less than the worn-out employees. Although the frenetic employees were predominantly less structurally impaired than the underchallenged employees, this difference was not statistically significant. This indicates similar structural impairments in the frenetic and the underchallenged burnout subtypes.

The *underchallenged employees* stated that they mainly felt insufficiently challenged at their workplace. Accordingly, the underchallenged employees correspond to the burnout subtype profile of Montero-Marin and García-Campayo [17]. In addition, the underchallenged employees were more cynical compared to the non-burned-out employees and the frenetic employees. These findings correspond with previous studies in which the underchallenged burnout subtype was particularly associated with cynicism [18,21,25]. In the present study, the underchallenged employees were also less professionally efficient, which is also in line with previous findings [26]. Apparently, not only high job demands but also feeling underchallenged at work can promote the development of burnout [25,122]. These study results are supported by previous studies in which boredom at work and burnout have been associated [123,124]. However, a corresponding burnout subtype, like the underchallenged burnout subtype, could often not be identified in previous studies [54,87,113,125]. Moreover, in a study on students, an underchallenged burnout subtype could be identified with the student version of the BCSQ-12 (BCSQ-12-SS: 19), but not with the student version of the MBI (MBI-SS: [126]) [26]. This suggests that the BCSQ-12 is a helpful questionnaire for investigating a burnout subgroup that has received little attention so far [26]. In addition, the underchallenged employees showed more passive coping strategies compared to the non-burned-out employees and the frenetic employees. This is reflected in the lower level of overload, the higher level of neglect, the lower level of work engagement and the lower level of social support among the underchallenged employees. Thus, the results of the present study are in line with previous study results in which the underchallenged burnout subtype was also more likely to be associated with passive coping strategies [26,63]. Compared to the study by Bauernhofer et al. [26], however, there was a significant difference between the underchallenged employees and the frenetic employees in terms of work engagement in the present study. In the study by Bauernhofer et al. [26], this difference was not significant. This could be because the individuals of the frenetic burnout subtype in this study had a stronger attitude of neglect and were therefore less engaged than the frenetic employees in the present study.

Finally, the *worn-out employees* stated that they neglected their work very much, thus corresponding to the burnout subtype profile of Montero-Marin and García-Campayo [17]. In line with previous findings [26,122], the worn-out employees had the least favourable burnout subtype profile compared to the non-burned-out employees, the frenetic employees and the underchallenged employees. The worn-out employees were the most exhausted, the most cynical and the least productive. Additionally, they were the most depressed, which corresponds to the description of Farber [127], who also characterized the worn-out subtype as depressive. Furthermore, these results are in line with previous study results, indicating that high levels of burnout are associated with depression [7,47–49,87]. The worn-out employees were also the least engaged, had the highest job demands but the least social support. Overall, the worn-out employees showed the strongest extent of passive coping mechanisms, thus being in line with previous study results [26,128]. Finally, the worn-out employees who showed the highest symptom load in the present study also had the strongest structural impairments. These results are in line with previous study results in which higher symptom load was associated with higher structural impairment [80,83,101]. According to the model of multilevel determinants of employee mental health [129], organizational, interpersonal and individual factors all contribute to the development of burnout [129]. Therefore, the high job demands, and the few social and individual resources among the worn-out employees of the present study may have contributed to their high levels of depression and burnout.

### Burnout as a developmental process

Overall, the trend towards a decrease in resources and an increase in demands and symptom load across the various burnout subtypes indicates a deterioration from the non-burned-out employee to the frenetic employee, the underchallenged employee to the worn-out employee. It seems that the burnout subtypes according to Farber [16] could represent different stages in the development of burnout. Leiter and Maslach [12] already formulated a developmental model of burnout, which begins with emotional exhaustion and develops through cynicism to reduced professional performance. In relation to the burnout subtypes according to Farber [16], the level of dedication [20,26,122] and the associated neuronal and physiological protective mechanisms in terms of the protective inhibition of self-regulation and motivation [130] could drive this transition process. According to Demarzo et al. [122], engaged people, as represented by the frenetic burnout subtype, may initially experience an increased level of costly physiological sympathetic activation and resource mobilization. To counteract the negative health effects of chronic over-engagement protective inhibition of self-regulation and motivation may set in to reduce the perceived amount available resources – as in the worn-out subtype – and thereby increase the likelihood of abandonment [122]. In the case of the underchallenged burnout subtype, the effort required for a task is low. However, the motivation of the underchallenged burnout subtype is also low, resulting in the limit of motivation being reached quickly. If the protective inhibition of self-regulation and motivation sets in, the individual´s motivation decreases, which ultimately leads to burnout [122].

## Limitations and implications

This study has several limitations that should be noted: First, the present sample was collected using a crowdsourcing platform. In literature, biases due to reduced attention when answering the items or increased psychological distress among crowdsourcing employees [131] are discussed as disadvantages of this form of data collection. Furthermore, self-selection processes, which are characteristic of online data collection, are likely. This could have led to individuals with greater digital affinity or more available time being overrepresented in the present sample. Moreover, the selection of the quota based on age, gender and place of residence also limits the representativeness of the sample, as other relevant sociodemographic variables, such as educational level, income, or migrant background, were not considered in the quota selection. Additionally, the sample consists exclusively of individuals from the general German working population. Since working conditions and job requirements can vary considerably across different occupational groups, no differentiated conclusion can be drawn regarding individual occupational groups or specific work contexts. Moreover, organizational factors also influence the risk of burnout. While high workloads, increased job demands, job insecurity or a low degree of control or reward increase the risk of burnout, support and fairness at the workplace have a protective effect against the development of burnout [132]. Finally, the generalizability of the results is limited due to the focus on a single national context. Cultural differences influence how mental health symptoms are perceived and expressed. While, for example, Western approaches to mental health tend to focus on individual intrapsychic experience, other cultures tend to emphasize somatic symptoms or interpersonal processes [133]. Besides, cultural differences influence response patterns in questionnaires. Depending on the cultural context, individuals tend to agree, exhibit socially desirable responses, or provide extreme responses [134]. Therefore, no conclusion can be drawn for other cultural contexts.

In addition, there is an increased risk of common method variance [135] in the present study through the use of self-assessment instruments. For this reason, future studies should use alternative sources for data collection [136].

Besides, the results of the exploratory factor analysis suggest that the original factor structure of the BCSQ-12 [21] is also preserved in the German working population. However, these findings should be further verified in future studies using confirmatory analyses. Another limitation concerns the four-cluster solution. Although it is theoretically plausible, it is based on an exploratory determination of the number of clusters. Due to the lack of stability analyses, the results should be interpreted with caution and replicated in independent samples.

Finally, the present study is a cross-sectional study, which does not allow any statements to be made about the stability of the burnout subtypes and their changes over time. To overcome these limitations, longitudinal data could be collected in future studies to gain a better understanding of the development of burnout subtypes, their determinants and consequences over time.

## Conclusion

To conclude, in the present study the BCSQ-12 was examined in terms of its internal consistency and its factorial and convergent validity using a sample representative of the German working population. This made the BCSQ-12 accessible for German-speaking countries and thus expanded its possibilities of application. Adopting a person-centred approach, in addition to a non-burned-out profile, three burnout subtypes – frenetic employees, underchallenged employees and worn-out employees – were identified, reflecting previous conceptualizations of burnout [16,17]. The identified burnout subtypes had individual burnout profiles and differed in terms of burnout symptoms, depressiveness, work engagement, job demands, social support and structural impairment. The present results emphasize the advantages of studying burnout using burnout profiles rather than individual scores for each of the three burnout dimensions [8]. Finally, the study results point to the need for tailored interventions [127].

## Supporting information

S1 Table English version of the BCSQ-12 and the German translation of the BCSQ-12. (PDF)
