# Supplementary material for: Burnout subtypes in the German working population: Differentiation by symptomatology, work-related factors and structural impairment according to the OPD
Source: PLoS One. 2026 Jul 6;21(7):e0352860. doi: 10.1371/journal.pone.0352860 (PMC13336201; doi:10.1371/journal.pone.0352860)
Supplement: S1 Table — (PDF) [file pone.0352860.s001.pdf]

S1 Table. English version of the BCSQ-12 and the German translation of the BCSQ-12

**BCSQ-12**	**German translation of the BCSQ-12**
Overload/Frenetic	
1. I think the dedication I invest in my work is more than what I should for my health.	1. Ich denke, ich investiere mehr in meine Arbeit als für meine Gesundheit gut ist.
4. I neglect my personal life when I pursue important achievments in my work.	4. Ich vernachlässige mein Privatleben, wenn ich wichtige Errungenschaften bei der Arbeit verfolge.
7. I risk my health when I pursue good resulty in my work.	7. Ich setze meine Gesundheit aufs Spiel, um bei meiner Arbeit gute Ergebnisse zu erzielen.
10. I overlook my own needs to fulfull my work tasks.	10. Ich übergehe meine eigenen Bedürfnisse, um die Arbeitsanforderungen zu erfüllen.
Lack of developement/ Underchallenged	
2. I would like to be doing another job that is more challenging for my abilities.	2. Ich würde gerne eine andere Tätigkeit ausüben, die meinen Fähigkeiten besser entspricht.
5. I feel that my work is an obstacle to the developement of my abilities.	5. Ich habe das Gefühl, dass meine Arbeit ein Hindernis für die Entwicklung meiner Fähigkeiten ist.
8. I would like to be doing another job where I can better develop my talents.	8. Ich hätte gerne einen anderen Job, bei dem ich meine Talente besser entfalten kann.
11. My work doesn´t offer me opportunities to develop my abilities.	11. Meine Arbeit bietet mir keine Möglichkeiten, meine Fähigkeiten zu entwickeln.
Neglect/Worn-out	
3. When things at work don´t turn out as well as they should, I stop trying.	3. Wenn die Dinge bei der Arbeit nicht so gut laufen, wie sie sollten, höre ich auf, es zu versuchen.
6. I give u in response to difficulties in my work.	6. Ich gebe auf, wenn ich Schwierigkeiten bei der Arbeit habe.
9. I give up in the face of any difficulties in my work tasks.	9. Ich gebe angesichts aller Schwierigkeiten bei meinen Arbeitsaufgaben auf.
12. When the effort I invest in work is not enoug, i give in.	12. Ich resigniere, wenn der Aufwand, den ich in die Arbeit investiere, nicht ausreicht.
